# Evaluating injuries and illnesses that occurred during the Yukon Quest International sled dog race, 2018–2020

**DOI:** 10.3389/fvets.2024.1356061

**Published:** 2024-02-27

**Authors:** Jenna C. Hattendorf, Michael S. Davis, Cristina M. Hansen

**Affiliations:** ^1^College of Veterinary Medicine and Biomedical Sciences, Colorado State University, Fort Collins, CO, United States; ^2^Department of Physiological Sciences, College of Veterinary Medicine, Oklahoma State University, Stillwater, OK, United States; ^3^Department of Veterinary Medicine, College of Natural Science and Mathematics, University of Alaska Fairbanks, Fairbanks, AK, United States

**Keywords:** Yukon Quest, orthopedic injuries, sled dog, dog mushing, canine sports medicine, veterinary medicine, canine orthopedic injuries, sled dog race

## Abstract

**Introduction:**

The purpose of this study was to evaluate medical record data from the 2018–2020 Yukon Quest International Sled Dog race to examine injury patterns and risk factors for dogs competing in multi-day ultra-endurance events. Specifically, we summarized injuries and illnesses that resulted in canine athletes being removed (“dropped”) from competition, and in orthopedic injuries diagnosed in both dropped and finished dogs.

**Methods:**

The records of 989 dogs that started the race were examined, but only records from dogs in teams that went on to finish the race were included, for a total of 711 records.

**Results and discussion:**

Three hundred and sixty five dogs (51.3%) were noted to have at least one abnormal finding in their veterinary medical record during the race. Orthopedic injuries were most common, and 291 injuries were ultimately diagnosed in 234 dogs (32.9%). Ultimately, 206 dogs (29%) were dropped from competition, for any reason. The most common reasons for dropping dogs were orthopedic injuries (156 dogs; 188 injuries), gastrointestinal illness (22 dogs), and cardiorespiratory disease (7 dogs). Most orthopedic injuries in dropped dogs occurred in the thoracic limb (*n* = 121 dogs; 151 injuries). Of those, injuries to the shoulder were most common (*n* = 77), followed by injuries to the carpus (*n* = 59), and injury to the pelvic limb (*n* = 32). Carpal injuries were the most prevalent injury diagnosed in dogs that went on to finish the race (71 of 85 injuries). Carpal injuries were the most prevalent injuries overall in 2018 (51%) and 2019 (52%). In 2020, shoulder injuries were most prevalent (27%), suggesting that trail conditions may have differed between years. The majority of dogs with an orthopedic injury ultimately were removed from competition (156 of 234, or 66.6%), but the likelihood of finishing the race with an injury depended on the type of injury sustained; 71 of 130 dogs (54.6%) with a carpal injury went on to finish the race, whereas only 9 of 86 dogs with a shoulder injury (10.5%) went on to finish. The results of this study can assist mushers and veterinarians in preparing for races, and in decision making during endurance sled dog races.

## Introduction

Ultra-endurance sled dog racing consists of teams of 12–16 dogs competing on wilderness trails over distances from 300–1,000 miles. These races last 2–12 days, during which time the teams pass through multiple checkpoints staffed by race officials and veterinarians. Dogs are examined at these checkpoints, and dogs are removed from the team (“dropped”) if the musher and/or veterinarian determine that the dog is unfit to continue in the race due to illness or injury. Typical race rules state that dogs cannot be replaced during a race; thus, dropping a dog results in a smaller team with less pulling power. In some instances, entire teams will elect to voluntarily withdraw or “scratch” from the race if circumstances suggest that continuing in the race is unlikely to be beneficial.

Several studies have been conducted to characterize the types of illnesses and injuries that occur in dogs during ultra-endurance racing. Von Pfiel et al. (1) documented orthopedic injuries sustained by dropped dogs during the 2011 Iditarod and associated injuries with various risk factors, including traveling speed and age. They documented that 43.3% of dropped dogs were dropped due to forelimb lameness, and 7.3% for hindlimb lameness, however this paper did not include data from dogs that finished the race. Many dogs (anecdotally, for example, those with carpal injuries) are diagnosed with mild to moderate orthopedic injuries, receive treatment and care on the trail, and are able to finish the race. One study did analyze records from both dropped and finished dogs competing in the Yukon Quest (2). That study characterized lameness as shoulder, carpal, or nonspecific, and examined records from 6 undefined locations during the race. That study documented forelimb lameness in 13.9% of all dogs (dropped and finished) competing in the Yukon Quest, but did not include a hindlimb lameness category. They also included diarrhea, cough, and “other disorder” reason for documenting dogs, but did not include other gastrointestinal or cardiorespiratory categories.

These previous studies provide a foundation for characterizing the types of illnesses and injuries that develop during ultra-endurance sled dog racing, but fail to provide a complete description of the risk factors for injury or for being dropped from a race. Therefore, the purpose of this study was to quantify and characterize the total number of orthopedic injuries and other illnesses incurred by all dogs competing in an ultra-endurance sled dog race, and also to examine the risk factors for dogs being dropped from that race in the hopes that this information will aid veterinarians who work with athletic dogs make decisions about their care.

## Methods

Veterinary medical record (hereafter “vet book”) data from 1,101 canine athletes that participated in the 1,000-mile Yukon Quest between 2018 and 2020 were compiled and analyzed. Each musher is allowed to have up to 16 dogs examined at pre-race veterinary checks, though they are allowed to start with 14 dogs. This provides them with 2 alternate dogs, should a dog incur an injury/illness in the weeks prior to the race start. During the race, mushers carry these vet books with them in their sleds as part of their mandatory gear. Upon arrival to each checkpoint, veterinarians read them and can follow-up with the health of individual dogs, and make entries about new exam findings in dogs.

The Yukon Quest Veterinary Team generally consists of 12 veterinarians and some veterinary support staff. Most of the veterinarians are general practitioners with an interest in working dogs, some are specialists in various fields, including sports medicine. Because of varying skill in the ability to diagnose orthopedic disease and the lack of diagnostic equipment on the trail, an exact cause of lameness is not always identified and vet book detail is sometimes lacking; i.e. some records state specifically that a shoulder is painful on extension vs. flexion or narrow the diagnosis to a particular musle, tendon, or joint. Some records simply state something akin to “shoulder injury.” Therefore, we did not categorize injury beyond joint in this paper.

For this study, a database was created and managed in Microsoft Excel that consisted of each athlete’s signalment, weight, body condition score (BCS) pre-race vital signs (temperature, pulse, respiratory rate) and physical examination findings, team notes, checkpoint physical examination findings, and the final disposition (finished, dropped, part of a scratched team, expired) of each dog.

**Figure 1:**
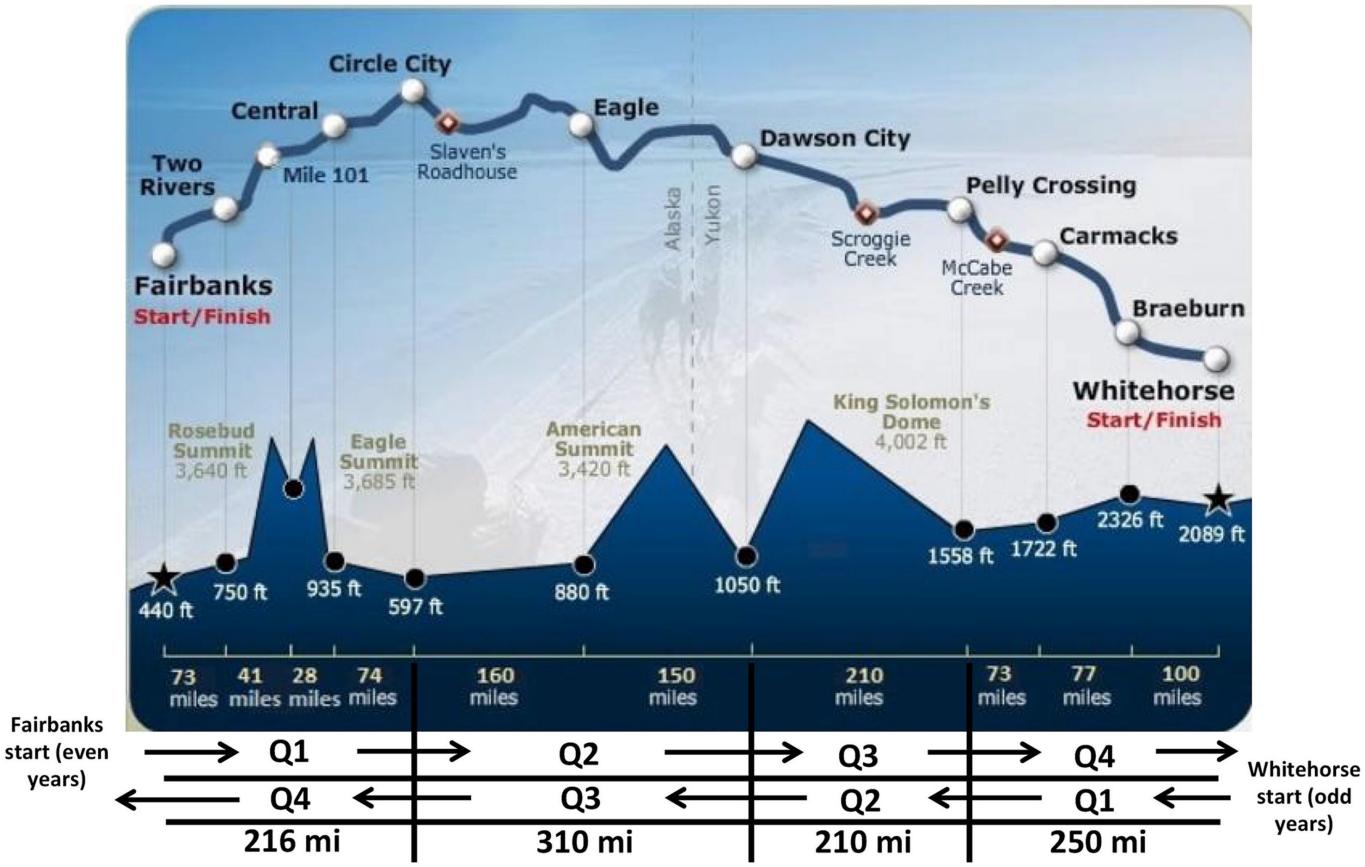
A map of the Yukon Quest trail superimposed over its elevation profile. The race alternates directions and starts in Fairbanks, AK (USA) in even-numbered years and in Whitehorse, YT (Canada) in odd-numbered years, so we cannot compare data checkpoint to checkpoint. We divided the race into quarters, as shown, to determine where dogs are likely to be dropped.

Each dog’s record was evaluated for the presence of an injury or illness that was mentioned at more than one checkpoint (indicating a persistent problem), required veterinary care, or caused the athlete to drop out of the race. These injuries were sorted into five categories: orthopedic, gastrointestinal (GI) disease/inappetence, cardiorespiratory disease, exertional rhabdomyolysis or other illness/injury. “Other Illness/Injury” included injuries or illnesses that occurred that did not fit the other categories, such as frost bite, harness rubs, and dogfight or other wounds. We also assigned a location of drop for each dog based on race quarter. Since the race alternates directions (Fairbanks to Whitehorse in even numbered years, and Whitehorse to Fairbanks in odd-numbered years), we cannot directly compare dogs dropped at each checkpoint. We divided the race into quarters based on milage as follows. In Fairbanks start years (even-numbered years): quarter 1 is from Fairbanks to Circle City (216 miles), quarter 2 is from Circle City to Dawson City (310 miles), quarter 3 is from Dawson City to Pelly Crossing (210 miles), and quarter 4 is from Pelly Crossing to Whitehorse (250 miles). In Whitehose start years (odd years) those quarters are reversed (Figure 1).

All statistical analyses were performed using R (3). Descriptive statistics (mean and standard deviation for continuous data, counts for categorical data) were calculated. We performed tests of equal proportions to determine whether the proportion of illnesses and injuries were different between years, and whether the location of dropped dogs (divided into race quarter) varied by year. Risk factors for individual dogs developing injuries or being dropped were assessed by fitting general linear models. Independent variables included demographic/trail factors (age, race direction, etc.) and dog illness/injury factors. Two models were evaluated: one assessing the affect of demographic/trail factors on the likelihood that a dog will develop an injury or be dropped from the race, and a second assessing whether or not dog illness/injury factors increase likelihood of being dropped. We did not include year and race direction in the same model, as those are directly related (in odd-numbered years the race direction is East to West, and vice versa). We further divided injuries into categories to determine which most likely would result in a dog being dropped. We only included dogs from teams that finished the race (i.e., excluding dogs that were dropped from teams that would go on to scratch or be withdrawn) in our analysis. An independent variable with *p* < 0.05 was considered significant.

## Results

### Demographics

One thousand one hundred and one (1101) dogs were examined at pre-race veterinary checks, which occur in the 2-weeks prior to the race start. 989 went on to start the race. We excluded records of dogs from teams that went on to scratch from the race, or were disqualified or withdrawn (278 dogs). The records from 711 dogs that started the race and were from teams that went on to finish were included in our analysis. The demographics (breed, age, sex, weight) of dogs included in this study are shown in Table 1.

**Table 1:** The average dog signalment 2018–2020.

	Average age	Age range	MI	FI	MC	FS	Average pre-race weight (kgs)	Alaskan Husky	Siberian Husky
2018	4.22	(1–8.5)	113	57	10	0	24.56	166	14
2019	4.22	(1–10)	211	147	18	2	24.27	350	28
2020	4.29	(2–8)	75	46	21	11	24.32	139	14
Total	4.24	(1–10)	399	250	49	13	24.38	655	56

### Incidence of injuries

**Figure 2:**
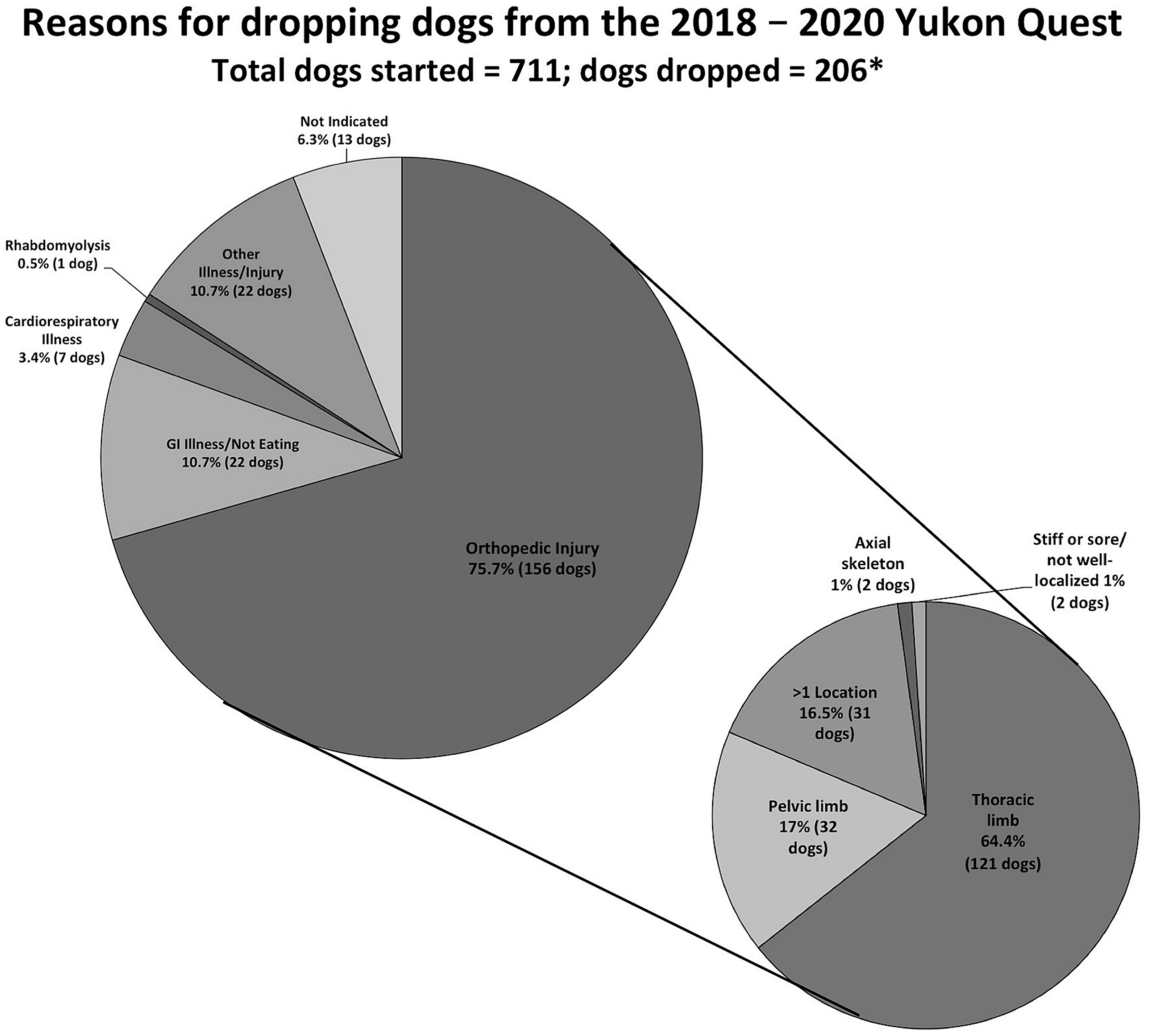
Reasons listed in veterinary medical records for dropping dogs during the 2018–2020 Yukon Quest. Orthopedic injuries are further characterized in the smaller pie. The records from 711 dogs from teams that would go on to finish the race are included. *Note that some dogs experienced multiple abnormalities so the number of reasons listed for dropping dogs exceeds the number of dropped dogs. Similarly, some dogs experienced multiple orthopedic injuries, so the number of injuries is greater than the number of dogs dropped for an orthopedic injury.

A total of 365 (51.3%) dogs experienced some type of injury or illness during the race. Two-hundred and six of those injured/ill athletes (56.4%) eventually dropped out of the race. In 2018, a total of 26.6% of dogs were dropped from the race (48 of 181); in 2019, 32% (121 of 378); and in 2020, 24.2% (37/153). These proportions were not statistically different between years (*p* = 0.14). The three most common reasons for dropping dogs during all 3 years analyzed were orthopedic injuries (156 dogs), GI illness or anorexia (22 dogs), and cardiorespiratory illness (7 dogs; Figure 2).

Among all dogs (those that finished and those that were dropped), orthopedic injuries were the most prevalent diagnosis in each year of the race (26.5% of dogs that started in 2018, 39.4% of dogs that started in 2019, and 24.2% of dogs that started in 2020), and most common reason for dropping dogs each year (32 dogs, 97 dogs, and 27 dogs, respectively). The overall number of orthopedic injuries diagnosed in all dogs is different between years (*p* = 0.0004).

**Figure 3:**
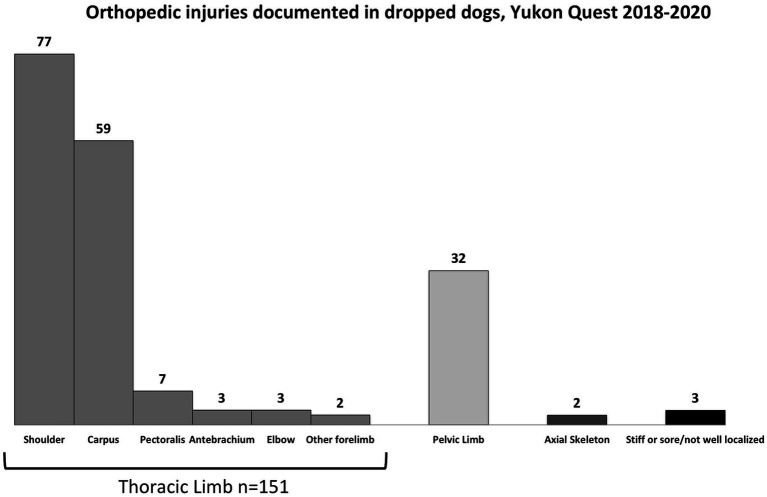
Proportions of carpal, shoulder, hindlimb, and other injuries diagnosed in dogs during the 2018–2020 Yukon Quest. The proportion of carpal injuries diagnosed was significantly different between years, as was the proportion of other injuries.

The most common site of orthopedic injury in all dogs (dropped and finished) varied year to year (Figure 3). In 2018 and 2019, the largest proportion of injuries occurred in the carpal joint, at 50.9 and 52%, respectively. In 2020, however, relatively more shoulder injuries (27%) than carpal injuries (19%) occurred. The proportion of carpal injuries among all dogs varied between years (*p* = 0.00002). The proportion of shoulder and hindlimb injuries did not vary (*p* = 0.75 and 0.39, respectively). The proportion of carpal injuries out of all orthopedic injuries varied by year (*p* = 0.007). The proportion of shoulder and hindlimb injuries out of all orthopedic injuries did not vary (*p* = 0.25 and 0.06, respectively).

**Figure 4:**
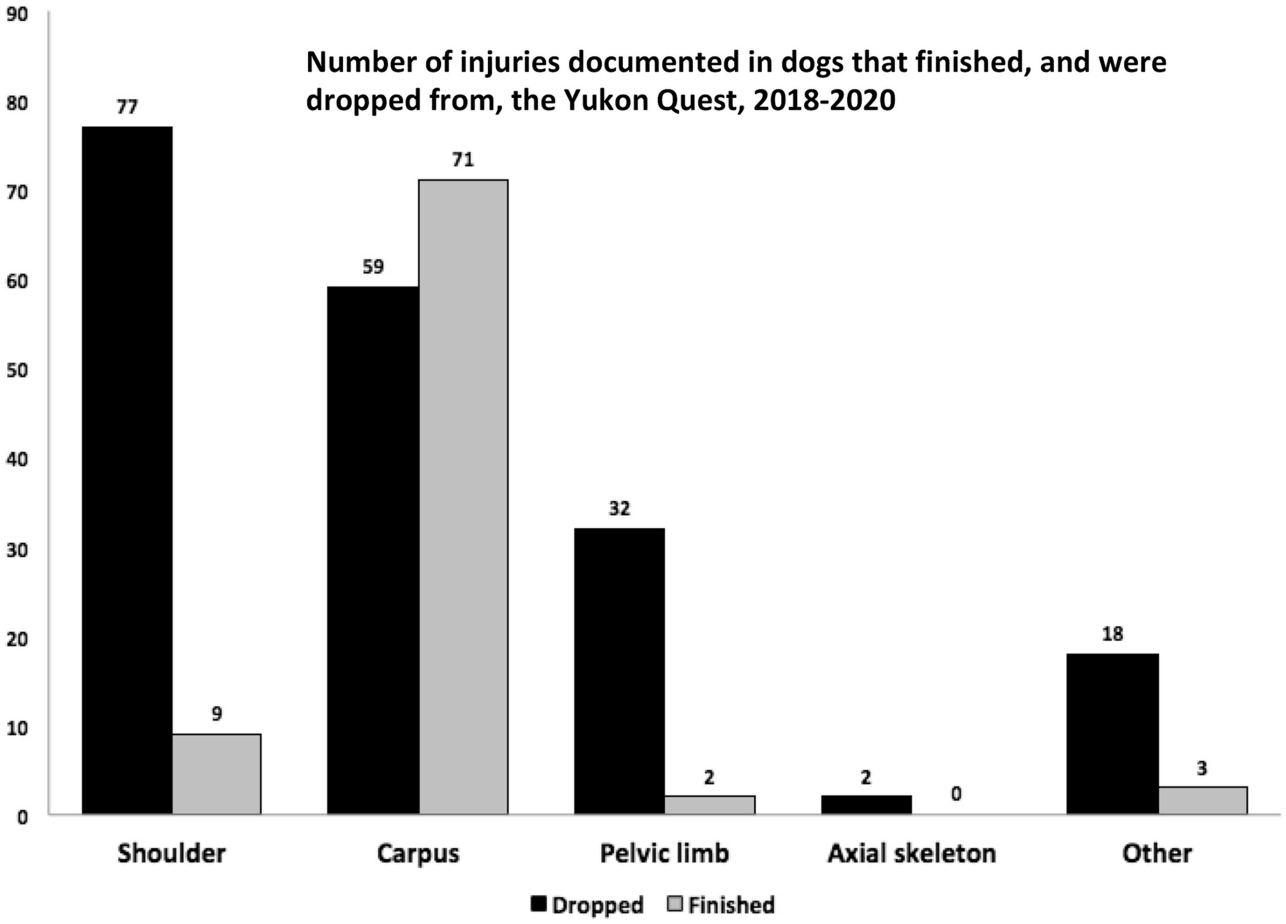
A summary of orthopedic injuries diagnosed in dropped dogs during the 2018–2020 Yukon Quest. Note that some dropped dogs were diagnosed with more than one injury, so there are more injuries documented here than there were dropped dogs due to injury in the previous figure.

Orthopedic injuries in dropped dogs were divided by localization (Figure 4). Injuries of the thoracic limb were most common in dropped dogs (151 injuries in 121 dogs.) followed by injuries to the pelvic limb (32). Two dropped dogs experienced an injury to their axial skeleton. Three dogs’ records reported that they were stiff/sore and could not localize an injury, or had an injury that was not well documented in the vet book (example, “lame” is all that is recorded). In the thoracic limb, shoulder injuries were most common in dropped dogs (77 injuries) followed by carpal injuries which occurred in 59 dropped dogs. Additional, less common thoracic limb injuries included those to the pectoral muscles (7 injuries), antebrachium (3 injuries), elbow (3 injuries), and other (2 injuries, including metacarpus and dewclaw injuries).

**Figure 5:**
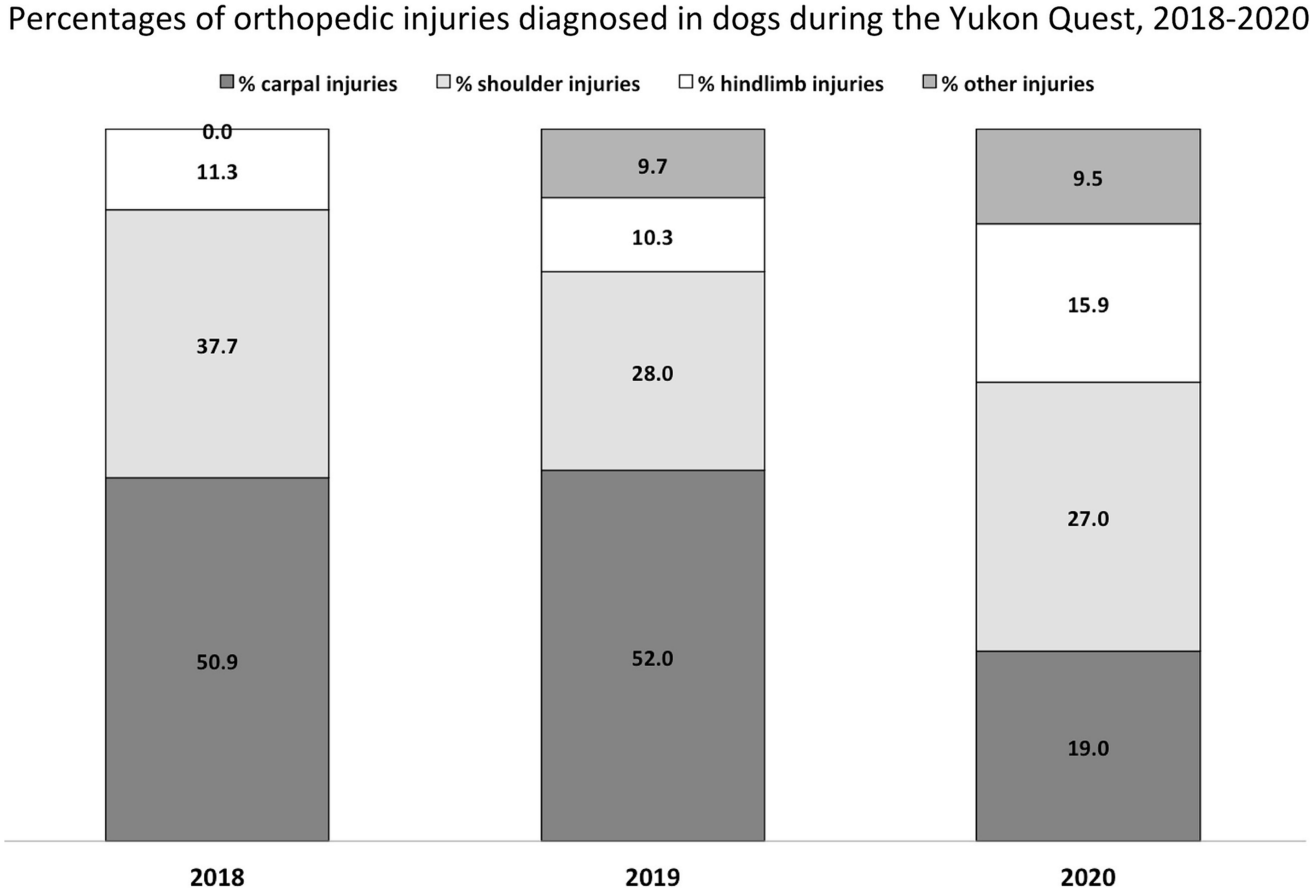
Orthopedic injuries documented in both dogs that finished, and were dropped from, the 2018–2020 Yukon Quest. Note that some dogs were diagnosed with more than one injury.

Seventy-eight dogs finished the Yukon Quest after sustaining some type of orthopedic injury. Some of those dogs experienced more than one injury, for a total of 85 injuries. These injuries were localized to compare to the injuries that occurred in dogs that were dropped from the race (Figure 5). In dogs that finished with an injury, the carpus was the most common site with 71 injuries (83.5%) noted, followed by 9 shoulder injuries (10.6%), 2 pelvic limb injuries (2.4%), and 3 other injuries (4%).

Out of the dogs that sustained a carpal injury, 54.6% (71 of 130) that sustained a carpal injury were able to finish the race, compared with only 9.4% of dogs (9 of 86) with shoulder injuries, 5.9% (2 of 34) pelvic limb injuries, and 0% (0 of 2) of axial skeleton injuries.

### Drop location

**Figure 6:**
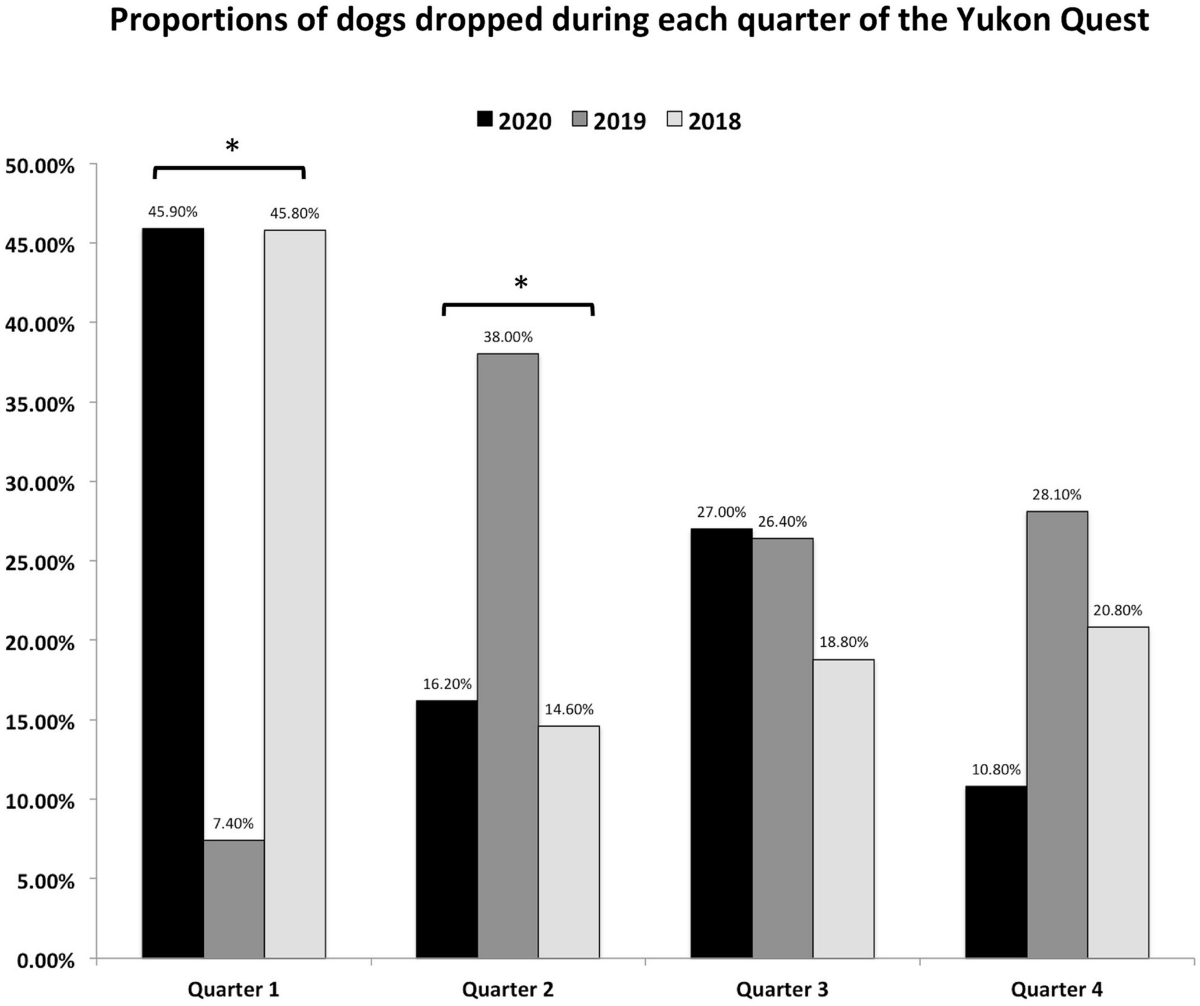
Proportions of dogs dropped during each quarter of the Yukon Quest. Note that the race alternates directions – starting in Fairbanks in even-numbered years and in Whitehorse in odd-numbered years. *Significant difference in proportions between groups (*p* < 0.05).

The trail was divided into four quarters to determine if there were specific portions of the trail where dogs were more likely to be dropped (Figure 6). Tests of equal proportions confirm that in even-numbered (Fairbanks-start) years, significantly more dogs are dropped during Q1 (*p* = 1.5 × 10^−9^), and in odd-numbered (Whitehorse-start) years, most dogs are dropped during Q2 (*p* = 0.001).

### Pre-race risk factor model results

The general linear model of dogs being dropped based on year (not race direction), breed, age, sex, and pre-race BCS found that Siberian huskies were less likely to be dropped (*p* = 0.02). Year (*p* = 0.61), age (*p* = 0.24), BCS (*p* = 0.08) and sex (*p* = 0.84 FS; 0.86 M; 0.28 MN) were not significant factors. When including direction (not year), Siberian huskies were still less likely to be dropped (*p* = 0.018). Direction appears to be a factor, with dogs less likely to be dropped in Fairbanks start years (*p* = 0.049). All other factors remain insignificant (age *p* = 0.24, BCS *p* = 0.08; FS *p* = 0.94, M *p* = 0.76, MN *p* = 0.2).

In our models testing effect of demographic and trail factors on the likelihood of dogs acquiring injuries (but not necessarily being dropped), when including year; neither year (*p* = 0.87), age (*p* = 0.45), pre-race BCS (*p* = 0.36), nor sex when compared to intact females (FS *p* = 0.81, M *p* = 0.37, MN *p* = 0.28) were significant factors. Siberian huskies are less likely to develop injury (*p* = 0.003). When including direction (rather than year), there is a strong effect of direction (*p* = 0.0001), with dogs more likely to be dropped when the race starts in Whitehorse. Siberian husky remains significant (*p* = 0.004), in that they appear to be less likely to be injured. Age (*p* = 0.42), pre-race BCS (*p* = 0.34), and sex (FS *p* = 0.86; M *p* = 0.49; MN *p* = 0.49) appear to not be significant factors.

### Intra-race risk factor model results

When examining injury/illness and likelihood of being dropped, dogs diagnosed with an orthopedic injury (*p* = 2 × 10^−16^), with a cardiorespiratory abnormality (*p* = 1.16 × 10^−5^), or with an “other” illness/injury (*p* = 6.86 × 10^−8^), were much more likely to be dropped. Dogs diagnosed with a gastrointestinal illness were not likely to be dropped (*p* = 0.21). Interestingly, dogs with rhabdomyloysis in this model were not more likely to be dropped, however, of the 2 dogs that developed rhabdomyolysis between 2018 and 2020, one was from a team that would later go on to scratch, so only 1 of these dogs was included in the statistical analysis, and that dog was dropped from the race.

Our models examining individual injuries show that dogs with injuries to the carpus (*p* = 2.6 × 10^−8^), shoulder (*p* < 2 × 10^−16^), pectoralis (*p* = 0.014), or hindlimb (*p* = 2.8 × 10^−9^) were much more likely to be dropped. Antebrachial (*p* = 0.29), elbow (*p* = 0.99), metacarpal (*p* = 0.63), axial skeleton (*p* = 0.98), or dewclaw (*p* = 0.99) injuries did not make it more likely for a dog to be dropped.

### Injury reoccurence

Thirty dogs that had an illness or injury went on to race a subsequent year. Five of those injuries reoccurred in the same location in four different dogs (13%). One dog had a reoccurring carpal injury, two dogs had a reoccurring shoulder injury, and one dog had both reoccur. Several of the dogs when dropped had multiple orthopedic localizations. In total, there were twenty-one carpal injuries and eleven shoulder injuries that did not reoccur. Dogs that experienced a hindlimb (4 dogs), pectoral (1 dog), dewclaw (1 dog), or other injury or illness (pneumonia, general soreness, cervical pain) did not experience it again.

## Discussion

Canine athletes are dropped for varied reasons during the Yukon Quest International Sled Dog Race. Some mushers choose to drop dogs when they are not enjoying the race or are not pulling well (“slack-lining”). Or, dogs can be dropped due to injury or illness.

The most common injuries incurred while racing are orthopedic. Unsurprisingly, dogs diagnosed with an orthopedic injury are less likely to finish a race than those without. However, not all orthopedic injuries are the same, and dogs have a good chance of recovering from and continuing a race if they experience a carpal injury. Therefore, timely and accurate diagnosis of lamemess by race veterinarians is paramount. Orthopedic injuries can be treated along the route with wraps, massage, heat and/or ice. However, the International Federation of Sleddog Sports bans the use of pain medications during competition and if needed the dog must be dropped (4).

Not only were orthopedic injuries very prevalent during our study period, they were consistently the most common reason for dropping a dog. This high prevalence of orthopedic injuries is also consistent with data from a previous study of multi-day ultraendurance sled dog racing (1). These combined data suggests that mushers should constantly assess their dogs’ gait for changes and report any suspected lameness to race veterinarians so the athlete can be properly evaluated. Identifying orthopedic injuries early allows for treatment to be initiated and may help the overall prognosis of the injury and prevent the need to drop the dog from the race. Some orthopedic injuries can also be prevented with endurance training prior to the race which helps strengthen bones, muscles, and tendons and stiffens cartilage and ligaments (5).

Thoracic limb injuries accounted for the vast majority of orthopedic injuries which is similar to previously reported injury localization in sled dogs and canine agility dogs (1, 2, 6). This is likely because the thoracic limb holds 60% of the bodyweight and is mainly responsible for stabilization as well as turning and steering and some propulsion (7). Meanwhile, the pelvic limb is mainly responsible for thrust and power and only bears 40% of the weight. The purposes of the limbs predispose the thoracic limb to injuries more than the pelvic limb.

The majority of injuries occurred at a joint, mainly the shoulder and carpal joints. Interestingly, dogs that compete in canine agility also have an increase in shoulder injuries (20%) but do not experience frequent carpal injuries (6%) in contrast to the sled dogs (8). Agility dogs also experience significantly more vertebrae injuries (6). Joints are uniquely susceptible to injury because they are under repetitive stress during exercise because the repetitive movements during exercise not only stress the joint but also the muscles that cross the joints and the ligaments needed for stabilization. Therefore, during a long endurance race such as the Yukon Quest the joints often are under great stress and can easily be injured. In addition, the joint is a common space for chronic disease processes such as osteoarthritis predisposing it to injuries during the race. Conditioning and training sporting dogs may help to prevent joint injuries, strengthen their muscles, and quicken athlete recovery if they do get injured (5). It is essential that dogs endurance train as well as strength train their muscles so that they are able to withstand long periods of exercise where they have to pull a sled. Most mushers condition their dogs by slowing increasing the mileage throughout the season which coincides with their race distances which often also get longer throughout the season. Conditioning also has physiologic effects such as decreasing resting heart rate, decreasing resting blood pressure, and increasing vascualization of the muscles allowing for more oxygen delivery (5). It is also important to note that too much conditioning may increase the likelihood of going into the race with a preexisting injury.

The brachial muscles (triceps brachii and biceps bracii) were included in our “shoulder” category since the race veterinarians in the field without diagnostic equipment are often unable to localize pain to a specific muscle, or do not record that specific information in the vet book. These muscles have an essential role in propelling the thoracic limb forward and supporting the limb during the weight bearing phase. The brachial muscles are therefore a crucial muscle group for movement. One study ultrasounded the shoulders of both dogs with shoulder pain and those without. Over 80% of the dogs ultrasounded had an abnormal finding. No correlation was found between clinical signs and abnormal ultrasound findings (9). In addition, there was a wide variety of shoulder abduction angles even in normal joints and fluid around the biceps tendon could not be related to pain in the shoulder. Therefore, in this study, ultrasound was unable to localize pain to a specific type of shoulder injury.

As noted in the results, the number of orthopedic injuries; specifically carpal injuries and “other” injuries sustained by dogs during the race was significantly different between years, but not their probability of being dropped once injured. This suggests that trail conditions, weather, etc. may impact the types of injuries that occur from year-to-year, but not the outcome of those injuries.

Our results are significant because they can help mushers and veterinarians decide whether a dog, based on the localization of the orthopedic injury, is statistically likely to finish, which may aid in decision-making regarding whether or not to drop a dog.

Carpal injuries were the most prevalent orthopedic injury followed by brachium and shoulder injuries. One hundred and forty-seven dogs experienced a carpal injury during the race and half of those athletes finished the race. In other words, 84% of athletes that finished with an orthopedic injury have a carpal injury. Meanwhile, only 11% of dogs that have a shoulder injury finished. This data indicates that an athlete with a carpal injury is more likely to respond to the treatment allowed during the race and be able to finish, compared to a dog with another orthopedic injury such as a shoulder or brachium injury localization. Based on this data, a dog experiencing an orthopedic injury other than a carpal injury is statistically unlikely to finish and a dog with a carpal injury has a 55% chance of finishing the race. This data allows veterinarians to give more informed recommendations to mushers about what types of orthopedic injuries should warrant dropping the dog from the race for recovery.

Cardiorespiratory disease also occurs on the trail, but can be difficult to properly diagnose without diagnostic equipment like ECGs, radiographs, and ultrasound available on the trail. Many trained sled dogs have “athletic heart syndrome,” which is a physiologic hypertrophy of the heart that leads to increased flow velocity through the aortic valve and a grade I-II/VI systolic murmur (10). As such, trail veterinarians need to be able to distinguish the physiologic heart murmurs that occur in many trained sled dogs from pathologic cardiac disease. Sometimes sudden cardiac deaths do occur on the trail (though they are often a diagnosis of exclusion – 10). Pneumonia has also been reported, and aspiration pneumonia is a leading cause of sudden death in sled dogs (11). We did document that 11 dogs were dropped from the Yukon Quest with cardiorespiratory illness in the 3 years studied, no dogs died from these causes. We also identified that being diagnosed with a cardiorespiratory abnormality makes it much more likely for a dog to be dropped from the Yukon Quest.

Another condition that mushers have to be aware of is exertional rhabdomyolysis (a.k.a. sled dog myopathy), which is another historical leading cause of death during dog sled races (12). Rhabdomyolysis is a serious condition that is caused by rapid muscle breakdown that occurs during high intensity exercise. This breakdown releases myoglobin into the bloodstream that travels to the kidneys for excretion and can lead to visible myoglobinuria (pigmenturia). Intuitively, and according to the human literature, rhabdomyolysis causes a marked hyperkalemia (13), as potassium is released from disintegrated skeletal muscle cells into the plasma. This can cause cardiac arrhythmias and sudden death. However, a study performed during the 2015 Yukon Quest found that canines with rhabdomyolysis experienced hypokalemia (12). Regardless, these clinical syndromes require significant and immediate veterinary care for the best clinical outcome. In addition, sometimes the myoglobin froms myoglobin casts that are large enough to obstruct renal tubular epithelial cells and can lead to an acute kidney injury in the longer term (after the race has finished), and these dogs are lost to follow-up (11). In 2015, 5 cases of rhabdomyolysis were diagnosed on the trail of the Yukon Quest (12), and the corresponding author remembers years on the Yukon Quest trail (prior to 2018, and not including 2015) with numerous cases of rhabdomyolysis. However, in the 3 years of data analyzed for this study, only 2 cases were documented, both were dropped from the race; neither dog died. The cause of rhabdomyolysis is still unknown, but it likely has some correlation with improper conditioning (e.g., not enough training miles or simulated races) leading up to a race (12).

Gastrointestinal illness and/or inappetence sometimes occur during the ultramarathon sled dog races and can be due to either stress or enteric infectious disease. In human medicine, ischemic colitis is recognized as a common cause of GI distress during endurance running (14, 15). Dogs undergoing short bouts of extreme exercise (up to 30 miles) have been shown to maintain visceral blood flow (16), but it is unknown whether visceral blood flow is maintained over many days of ultramarathon racing where dogs are averaging more than 100 miles per day. In addition, both *Salmonella* and *Clostridium* species numbers have been documented to increase in the feces of dogs during races, however they were present in both dogs that experienced diarrhea and those who did not and therefore they cannot be implicated as the cause of disease (17). There are anecdotal reports that during warm weather, dogs are more likely to have a viral or bacterial outbreak that causes diarrhea; and warm/spoiled food or drop bags are sometimes implicated. We showed that being diagnosed with a gastrointestinal abnormality (most commonly diarrhea) did not increase the likelihood that a dog will be dropped from the Yukon Quest. In fact, as our data shows, many dogs diagnosed with diarrhea early in the race experience resolution of it and go on to finish in good health.

We examined whether any demographic factor (breed, sex, age) increased the likelihood of a dog being dropped from the Yukon Quest, in addition to injury and illness. We found that Siberian Huskies are less likely to be dropped from the Yukon Quest as well as are less likely to develop injury than Alaskan huskies. The Siberian Husky is an AKC registered breed with ancient roots that has historically been used to pull sleds. The Alaskan husky is not an AKC recognized breed and has genetics from other breeds in it’s recent history. Most modern long distance race teams are made of the smaller, leaner, faster Alaskan huskies. It may be that Siberian Huskies are less likely to be dropped from races because they tend to run at slower speeds, are larger bodied, or have genetics that protect them from injury/illness on the trail. In addition, in this study only a select few number of mushers had teams with Siberian Huskies and they always made up the whole team. The way that these specific mushers cared for their dogs may also have contributed to the Siberian Huskies having a lower likelihood of being dropped during the race.

While the data stated can be generalized to other endurance sled dog races, there are some unique obstacles during the Yukon Quest trail such as the race direction as well as the trail conditions. Since the Yukon Quest changes direction in odd- and even- numbered years, we cannot compare checkpoints year-to-year. However, we divided the race into quarters to determine where along the race more dogs are dropped. We determined that when the race starts in Fairbanks, more dogs are dropped early, in the first quarter of the race. This makes sense, as the most notorious sections of trail (Eagle Summit) occurs during that section – Eagle Summit has steep elevation grades and is subject to blizzard conditions often during the race. It may be likely that mushers with inexperienced dogs are likely to drop them during this section of race. When the race starts in Whitehorse, Eagle Summit is later in the race, and those inexperienced dogs may have been dropped anywhere prior in the race, without that early pressure to do so. The dogs that make it to Eagle Summit in odd years are likely seasoned veterans and are less likely to be dropped. In odd-numbered years, more dogs are likely to be dropped in the second quarter of the race, which includes the longest unsupported stretch of trail (Pelly-Crossing to Dawson City – 210 miles). Similarly, mushers may feel compelled to leave inexperienced or young dogs behind prior to starting this long remote stretch of trail.

The Yukon Quest trail, weather, and musher decisions also play a role in the race. The trail often requires teams to make sharp turns and change direction to stay on course, which may predispose dogs to orthopedic injuries. In addition, some years there may be more ice on the course that causes slipping and prevents the dogs from gaining traction during the tight turns. Sometimes during the race, teams face snow storms and a large snow fall may cover the groomed trail. In this case, the dogs may steer away from the trail and punch through deep snow, causing a thoracic limb injury. Anecdotally, some mushers report that not using necklines reduces the incidence of front end injuries. The individual choices of the musher can also make a team more or less susceptible to injuries. The trail and weather conditions as well as the judgement of the musher are all factors that influence whether a dog will be dropped from the race. The data and conclusions that we provide in this paper not only add to the body of knowledge about sport dogs, injuries, and predisposing factors, but also will help guide mushers and race veterinarians in their decision making during races.

### Future directions

The database created from this research is filled with valuable information. One future direction that we would like to explore is how weather during the race can impact the prevalence of orthopedic injuries and the localizations of those injuries. The type of snow, fresh or packed, may also have an impact on the types of injuries seen in athletes. Weather may also impact the prevalence of GI disease as some pathogens thrive at warmer temperatures and others may be more easily transmitted when the dogs are in closer proximity when it’s cold.

## Data availability statement

The data analyzed in this study is subject to the following licenses/restrictions: This dataset comes from veterinary medical record data that is not public. Requests to access these datasets should be directed to CH, cmhansen@alaska.edu.

## Author contributions

JH: Data curation, Investigation, Writing – original draft, Writing – review & editing. MD: Formal analysis, Methodology, Writing – review & editing. CH: Conceptualization, Formal analysis, Methodology, Supervision, Visualization, Writing – review & editing.
